# Stopping “transient ischemic attacks” by antiplatelet withdrawal

**DOI:** 10.1186/s42466-021-00117-0

**Published:** 2021-04-01

**Authors:** Lina Palaiodimou, Aikaterini Theodorou, Stefanos Lachanis, George P. Paraskevas, Matilda Papathanasiou, Christina Zompola, Konstantinos I. Voumvourakis, Georgios Tsivgoulis

**Affiliations:** 1 Second Department of Neurology, National and Kapodistrian University of Athens, School of Medicine, “Attikon” University Hospital, Rimini 1, Chaidari, 12462 Athens, Greece; 2 Iatropolis Magnetic Resonance Diagnostic Centre, Athens, Greece; 3 Second Department of Radiology, National and Kapodistrian University of Athens, School of Medicine, “Attikon” University Hospital, Athens, Greece; 4 Department of Neurology, The University of Tennessee Health Science Center, Memphis, TN USA

**Keywords:** Transient ischemic attacks, Cortical superficial siderosis, Cerebral amyloid angiopathy, Antiplatelet treatment

## Abstract

**Introduction:**

Transient ischemic attack (TIA) is considered to be an important risk factor for the development of ischemic stroke and requires complete etiopathogenic evaluation and prompt initiation of secondary prevention treatment. In addition, an accurate differential diagnosis should be performed in order to exclude other disorders mimicking TIA.

**Methods:**

In this case report, we describe the clinical and neuroimaging evaluation and the differential diagnosis of a patient with suspected crescendo TIAs.

**Results:**

A 79-year-old man presented with recurrent episodes of right-sided numbness over the past 7 months, despite different single and dual antiplatelet therapies that were sequentially prescribed for suspected TIAs. Brain MRI revealed cortical superficial siderosis, symmetrical periventricular leukoencephalopathy and enlarged perivascular spaces. Cerebral amyloid angiopathy was considered in the differential diagnosis of the patient. Antiplatelet withdrawal was recommended and led to complete remission of the patient’s transient focal neurological episodes (TFNE) that were initially misdiagnosed as TIAs.

**Discussion:**

Cortical superficial siderosis has been implicated as a key neuroimaging feature of cerebral amyloid angiopathy, a diagnosis which can be supported by the additional radiological findings of symmetrical white matter hyperintensities and enlarged perivascular spaces. Antiplatelet treatment in patients with cortical superficial siderosis may increase the frequency and severity of TFNE, while it increases exponentially the risk of intracerebral hemorrhage. The present case highlights that recognition of cortical superficial siderosis is crucial in the management of patients presenting with transient focal neurological symptoms that can be misdiagnosed as recurrent TIAs.

## Introduction

Transient ischemic attacks (TIAs) are defined as sudden-onset, brief episodes of focal loss of brain function due to underlying cerebrovascular disease and typically last less than 24 h [1]. TIAs are considered to be an important risk factor for the development of an ischemic stroke. For that reason, patients presenting with TIAs should undergo a complete etiopathogenic evaluation and appropriate secondary prevention treatment should be promptly initiated [2]. However, an accurate differential diagnosis should initially be performed in order to exclude other disorders mimicking TIAs [3].

## Methods

We present the clinical and neuroimaging evaluation of a patient who was initially diagnosed with crescendo TIAs and further describe the differential diagnosis of this case.

## Case report

A 79-year-old man with no previous medical history presented to an outside Institution with recurrent episodes of right sided numbness. Different single and dual antiplatelet therapies were sequentially prescribed for suspected crescendo TIAs over the past 7 months, without any impact on the recurring neurological symptoms. On the contrary, clinical deterioration was reported with substantial escalation in the frequency, duration and severity of the episodes. The patient subsequently received a trial of antiepileptic treatment (levetiracetam 1000 mg bid), despite that repeat electroencephalograms (EEG) were normal. Furthermore, magnetic resonance angiography of extra- and intracranial arteries, echocardiography and 24-h Holter electrocardiogram recording were negative. The patient was referred to our Institution for re-evaluation of his symptoms.

**Fig. 1:**
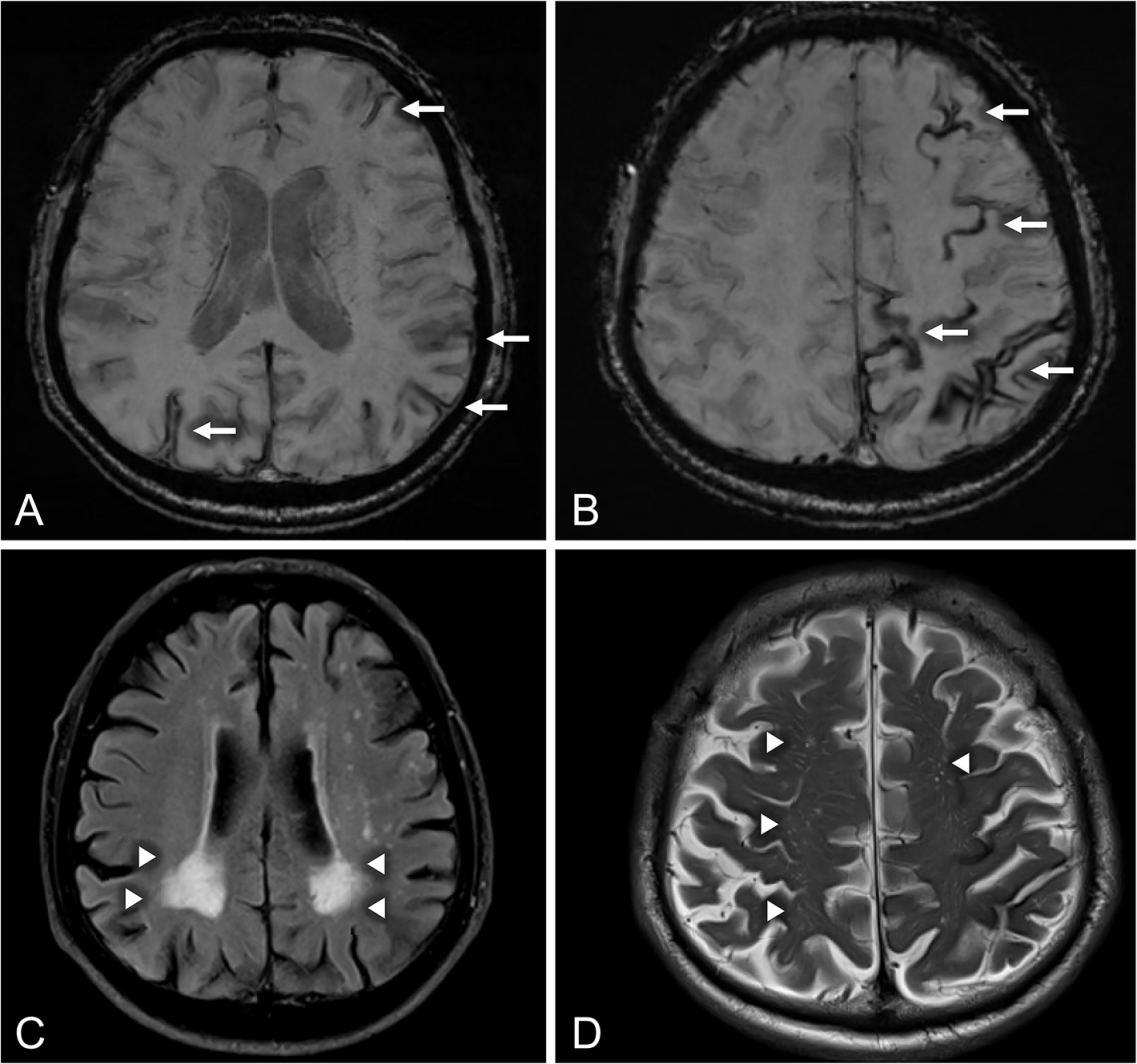
Neuroimaging findings in a patient with transient focal neurological episodes. Legend: Axial brain MRI with susceptibility-weighted imaging sequence showing rims of low signal coating the sulci of both cerebral hemispheres with left predominance, suggestive of cortical superficial siderosis (Panel **a**-**b**, arrows). Axial brain MRI with fluid-attenuated inversion recovery sequence showing symmetrical periventricular white matter hyperintensities with occipital predominance of Fazekas grade 3 (Panel **c**, arrowheads). Axial brain MRI with T2-weighted imaging showing enlarged perivascular spaces in centrum semiovale (Panel **d**, arrowheads)

**Fig. 2:**
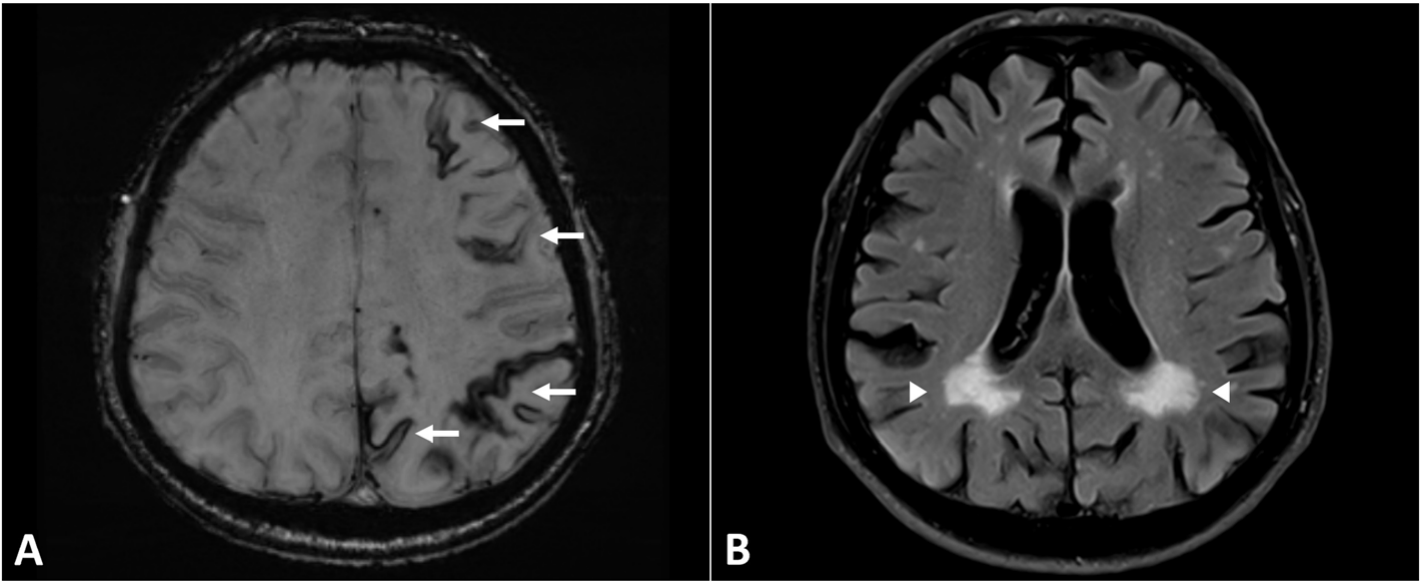
Follow-up neuroimaging findings at 1 year. Legend: Axial brain MRI with susceptibility-weighted imaging sequence showing that cortical superficial siderosis has been slightly restricted compared to the baseline MRI (Panel **a**, arrows). Axial brain MRI with fluid-attenuated inversion recovery sequence showing stabilization without further expansion of the symmetrical periventricular white matter hyperintensities compared to the baseline MRI (Panel **b**, arrowheads)

Brain magnetic resonance imaging (MRI) was performed revealing rims of low signal in susceptibility-weighted imaging sequence coating the sulci of predominantly the left hemisphere, suggestive of cortical superficial siderosis (CSS; Fig. 1, Panel A-B, arrows). Posterior fossa was spared. No microbleeds were disclosed. Symmetrical periventricular white matter hyperintensities (Fazekas grade 3, Fig. 1, Panel C, arrowheads) and enlarged perivascular spaces (Fig. 1, Panel D, arrowheads) were present in fluid-attenuated inversion recovery sequence. No lesions with restricted diffusion suggestive of ischemia were noted in diffusion-weighted imaging sequence. Based on those neuroimaging findings, the diagnosis of possible cerebral amyloid angiopathy (CAA) according to the modified Boston criteria was postulated [4]. Antiplatelet withdrawal was recommended and led to complete remission of the patient’s transient focal neurological episodes (TFNE) that were initially misdiagnosed as TIAs. Antiepileptic treatment was also discontinued 8 weeks later, since no further symptoms occurred and repeat EEGs were normal. Since then, the patient has been followed both clinically and radiologically. Follow-up brain MRI scans were performed at 6 and 12 months, excluding additional macro- and micro- intracerebral hemorrhages (Fig. 2), and the patient has remained symptom-free for the past 15 months.

## Discussion

CSS has been implicated as a key neuroimaging feature of CAA, a diagnosis which can be supported by the additional radiological findings of symmetrical white matter hyperintensities and enlarged perivascular spaces [5]. For that reason, CAA was also included in the differential diagnosis of the patient, despite that no cognitive impairment was identified. However, TFNE which are defined as recurrent, stereotyped symptoms usually lasting for several minutes, have also been documented as part of the clinical manifestations of CAA [6]. In addition, TFNE are shown to correlate well with the neuroimaging findings of CSS and disseminated CSS (in 4 or more sulci), which were present in our patient [7]. TFNE with predominantly negative symptoms, such as our patient’s numbness, have been described as “TIA-like” episodes and are shown to be equally common to those with positive symptoms (“aura-like”) [7]. The pathophysiological correlation between TFNE and CSS may lie in focal seizure activity or cortical spreading depression due to cortical irritation, but, additionally, local vasospasm due to accumulating blood-breakdown products may lead to predominantly negative symptoms [8]. In the case of our patient, an epileptic origin of the symptoms may have been possible, despite the normal EEG. Symptomatic seizures could occur for a limited time when caused by an acute bleeding event and then stop after consolidation of the bleeding when antiplatelets were withdrawn. Conversely, the addition of antiplatelets in patients with CSS may escalate the frequency and severity of TFNE, while it increases exponentially the risk of intracerebral hemorrhage [9]. It is important to differentiate between TFNE and TIAs by performing brain MRI with susceptibility-weighted imaging sequence.

In conclusion, the present case highlights that recognition of CSS is crucial in the management of patients presenting with transient focal neurological symptoms that can be misdiagnosed as recurrent TIAs, leading to initiation and escalation of antiplatelet treatment and possibly symptom worsening due to incident micro- and macro-bleeding.

## Data Availability

All data are presented in the manuscript.

## Abbreviations

TIA: Transient ischemic attack
EEG: Electroencephalogram
MRI: Magnetic resonance imaging
CSS: Cortical superficial siderosis
TFNE: Transient focal neurological episodes
CAA: Cerebral amyloid angiopathy
